# Awareness of forensic anthropology in Switzerland: a survey among forensic practitioners, police, and prosecutors

**DOI:** 10.1007/s00414-023-03116-9

**Published:** 2023-11-15

**Authors:** Inga Siebke, Claudine Abegg, Tony Fracasso, Negahnaz Moghaddam, Zuzana Obertová

**Affiliations:** 1 Zurich Forensic Science Institute, Zurich, Switzerland; 2 Unit of Forensic Imaging and Anthropology, University Centre of Legal Medicine Lausanne-Geneva, Lausanne University Hospital and University of Lausanne, Lausanne, Switzerland; 3 Unit of Forensic Medicine, University Centre of Legal Medicine Lausanne-Geneva, Lausanne University Hospital and University of Lausanne, Lausanne, Switzerland; 4 Unit of Forensic Medicine, University Centre of Legal Medicine Lausanne-Geneva, Geneva University Hospital and University of Geneva, Geneva, Switzerland; 5 Swiss Human Institute of Forensic Taphonomy, University Centre of Legal Medicine Lausanne-Geneva, Lausanne University Hospital and University of Lausanne, Chemin de la Vulliette 4, CH – 1000, 25 Lausanne, Switzerland; 6 Centre for Forensic Anthropology, School of Social Sciences, The University of Western Australia, Perth, Australia

**Keywords:** Forensic anthropology, Human remains, Questionnaire, Forensic collaboration, Legal medicine, Facial image comparison

## Abstract

Forensic anthropology (FA) as a specialized discipline has been practised in multi-lingual Switzerland for over a decade. A variety of expertise regarding osteological assessments as well as facial image comparison (FIC) is provided by different centres. Nevertheless, information is lacking about the awareness of FA and its benefits for forensic investigations among forensic stakeholders. Therefore, a survey was sent to Swiss anthropologists (AN) and related professions (police officers, prosecutors, and forensic pathologists) to assess three main aspects: (1) the experience of working (biological/forensic) anthropologists within FA; (2) how FA is perceived by other professions within the legal system; and (3) identify gaps (if any) in understanding of FA with the aim to suggest avenues for improvement if necessary. The results show that awareness of FA varies by occupation and cantonal regions. In areas where close collaborations between forensic anthropologists (FAs) and other stakeholders have been formally established, be it with focus on osteological analyses or FIC, the awareness of FA competencies was superior to areas where this was not the case. An overwhelming majority of forensic actors expressed interest in continuing education related to the role of FA. These findings indicate that facilitation of communication and collaboration leads to improvement in the awareness of the competencies of FAs and their contribution to forensic investigations.

**Supplementary Information:**

The online version contains supplementary material available at 10.1007/s00414-023-03116-9.

## Introduction

**Fig. 1:**
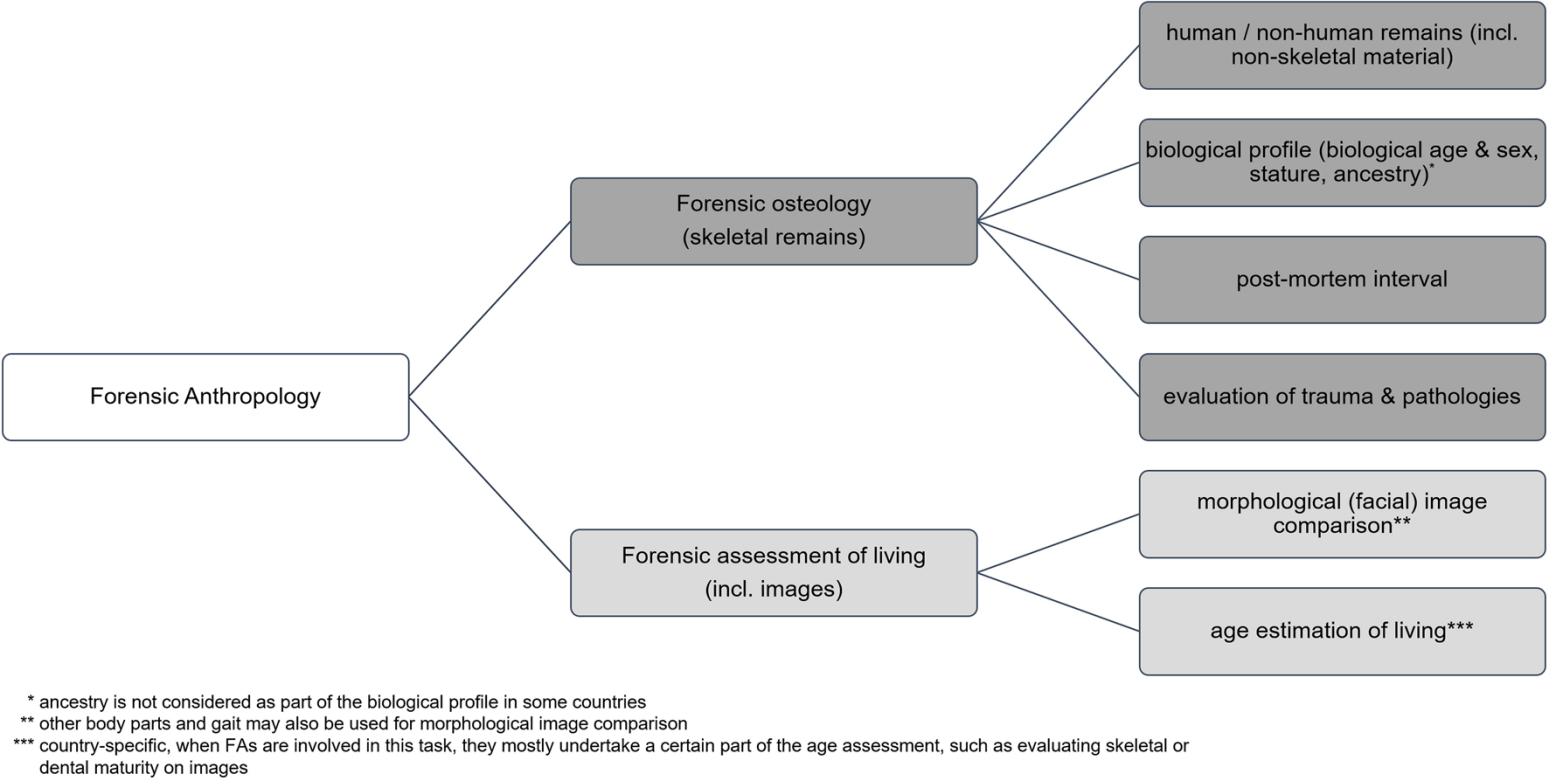
Main areas of work and responsibilities of forensic anthropologists

Forensic anthropologists (FAs) take on numerous roles (Fig. 1) since the tasks allocated to them are commonly dictated by the needs of local law enforcement agencies, as well as forensic and judiciary institutions. These tasks can vary from the analysis of human skeletal remains to facilitate identification, skeletal trauma analysis, visual identification of persons, or being part of Disaster Victim Identification (DVI) teams [1–4].

Kranioti and Paine [1], in their overview of forensic anthropologists in Europe from 2011, have mentioned diverse employment situations for FAs within Europe, mainly freelance actives, and some employment in academia or governmental institutes. While pointing out that among the 18 European countries evaluated in their study, most of the cases involving skeletal remains were handled by forensic pathologists and/or anatomists, and only in two countries physical anthropologists are reported to be involved in skeletal evaluation [1]. They argue that this division of tasks may be due to a lack of specific educational pathways for FAs; as anatomists and forensic pathologists have been the traditional professions dealing with forensic requests concerning skeletal remains [1]. A similar diversity in the involvement of FAs in forensic casework has been reported in a survey by Obertová et al. [5] from 2019. However, there has been some progress since among 28 studied countries, 20 countries reported that anthropologists (AN) were involved to at least some extent in cases of skeletonized remains, while in Austria and Finland only FAs, not forensic pathologists, were handling such cases. Switzerland was not included in the [1] survey, but at that time, in contrast to many other European countries, AN had already been working on forensic cases, holding positions at the Unit of Forensic Imaging and Anthropology at the University Centre of Legal Medicine in Lausanne/Geneva (CURML), and at the Department of Physical Anthropology (DPA) at the Institute of Legal Medicine in Bern.

Switzerland is divided into 26 cantons. Besides the federation, each canton has its own cantonal laws, judiciary, and police corps within a federal system. Although Switzerland has a population of only 8.7 million [6], there are seven institutes of legal medicine (in alphabetical order: Aarau, Basel, Bern, Chur, CURML in Lausanne and Geneva, St. Gallen, Zurich). As mentioned previously, forensic anthropology can be found in Lausanne/Geneva and in Bern, while the Institute of Forensic Medicine in Zurich has a collaboration agreement with the AN based at the Zurich Forensic Science Institute since 2020.

The CURML covers the forensic needs of French-speaking Switzerland (mainly the cantons of Vaud, Valais, Geneva, Fribourg, Neuchâtel, and the Jura). The focus of FA at the CURML lies mostly with forensic casework and research. The Department of Physical Anthropology at the Institute of Forensic Medicine in Bern is also involved in forensic casework. However, as described by Indra and Lösch [7] the main investigations concern archaeological skeletal remains and only a smaller proportion relates to forensic contexts. The Zurich Forensic Science Institute (FOR), an independent governmental police institute, is responsible for crime scene investigations and provides forensic specialist consultations to the police of mainly German-speaking cantons. The anthropologists are based at the Unit of Visual Identification of Persons (VIP), with their casework encompassing mainly facial image comparison (FIC) and more recently osteological analyses (collaborating with the Institute of Forensic Medicine at the University of Zurich).

Apart from the FAs working at the above-mentioned institutions, there are freelance AN and AN employed within archaeological excavation firms. The primary casework of these two groups concerns archaeological remains, with occasional involvement in forensic casework. There is no specific higher degree in forensic anthropology offered at Swiss universities, although AN/FAs at universities in Basel, Bern, Lausanne and Geneva, and those from the FOR in Zurich, do provide individual lectures for under- and post-graduate courses in medicine, law, and archaeology.

Prior studies have shown that acceptance and knowledge of FA on the national level varies widely [1, 5, 8–14]. For example, just recent and very limited involvement of FAs in relevant forensic investigations are reported from Finland [14]; for Sweden, only some FAs involvement in casework mainly depending on the knowledge/personal contacts of the crime scene investigator or forensic pathologists in charge are stated by Alfsdotter [8] and Alfsdotter et al. [13], whereas clear collaboration structures for FAs in Denmark exist and different aspects of FA are practised at three different departments of forensic pathology [12]. Compared to these three countries, the Swiss situation of forensic anthropology and forensic anthropologists seems to be most comparable with the Swedish situation. Despite the varied employment opportunities for AN in Switzerland, little is known about the knowledge of their role regarding human identification and other aspects of forensic anthropological expertise among other actors involved in forensic casework, such as police officers (PO), prosecutors (PR), and forensic pathologists (FP).

The aim of this study was therefore to survey the state of FA in Switzerland and more specifically the perception of the role of FA both within the discipline of anthropology, and among other actors of legal proceedings likely to engage with FAs, namely police officers, prosecutors, and forensic pathologists. The objectives were to acquire knowledge about (1) the experience of practitioners of FA in Switzerland and the methods used in forensic analyses; (2) how FA is perceived by other professions within the legal system; and (3) identify gaps (if any) in the understanding of the role of FA and suggest potential avenues of improvement in areas of need.

## Materials and methods

### Materials

Four anonymous questionnaires were designed for this survey, using the LimeSurvey platform (LimeSurvey Community Edition, Version 3.28.19+220712), access to which was provided through the University of Geneva, Switzerland. Each questionnaire targeted a specific profession: anthropologists (AN), police officers (PO), prosecutors (PR), and forensic pathologists (FP). The questionnaires were designed in English, and then translated into three of the official Swiss languages [15]: German, French, and Italian. The English version of the questionnaires can be found in Supplementary Material 1.

At the beginning, each participant was asked to name the canton of their primary employment. No identifying information beyond the main canton of employment was collected. The questionnaires consisted of eight to eleven questions and were organised into three main parts: expertise of FA, awareness of the role of FA, and education and training. For each profession, the survey questions were adapted to the field of expertise; however, the content of the questions was similar to ensure comparability across the different professions.

### Methods

To reach the members of the various professions targeted by the questionnaires, invitations to distribute the survey were sent by email to various national working groups (WGs) in 2022. The WGs included the Swiss Anthropological Society (SGA/SSA), police WGs in the individual cantons and at the FedPol (the Swiss Federal Police), as well as the prosecutors’ WG. Forensic pathologists were approached through their respective institutions with emails sent to all institutes of legal medicine in Switzerland.

The questionnaires were available online for two months, after which time all results were exported as Microsoft Excel files. Descriptive statistics were performed using Microsoft Excel using percentages of responses compared among the professions and, where relevant, among cantons of employment grouped by official language. The cantons of employment or canton groups were classified as follows

I. DEC—cantons with German as the official language (Argovia, Appenzell Outer-Rhodes, Appenzell Inner-Rhodes, Basel, Basel District, Glarus, Lucerne, Nidwalden, Obwalden, Schaffhausen, Schwyz, Solothurn, St Gallen, Thurgau, Uri, Zug, Zurich);
II. FRC—cantons with French as the official language (Geneva, Vaud, Neuchâtel, Jura);
III. MLC—cantons with two or more official languages (Bern, Fribourg, Valais, Grisons), the Canton of Ticino, where Italian is the official language, was also included in this group.

The responses of police officers (PO), prosecutors (PR), and forensic pathologists (FP), regarding their knowledge of different methodologies potentially associated with FA (question 7) were grouped into four main categories: general (morphological, metric), specialized (FIC, facial approximation), technical (imaging, histology), and analytical (dating, isotopes).

## Results

### Response rate

Overall, there were 188 responses, 16 responses from anthropologists (AN), 65 from police officers (PO), 80 from prosecutors (PR), and 17 from forensic pathologists (FP). Table 1 shows the distribution of responses by canton group including the proportion of the Swiss population living within these cantons, which was used as a proxy to assess the response rates. According to this proxy comparison the responses from AN and PO in DEC and from FP in MLC (a single response) were underrepresented.

**Table 1:** Distribution of responses by cantons grouped according to official languages; *AN*, anthropologists; *PO*, police officers; *PR*, prosecutors; *FP*, forensic pathologists

Official language In canton/ Profession	AN	PO	PR	FP	Percentage population living in canton group
Canton group	*n* (%)	*n* (%)	*n* (%)	*n* (%)	
German (DEC)	5 (31)	19 (29)	52 (65)	10 (59)	56%
French (FRC)	5 (31)	26 (40)	12 (15)	6 (35)	18%
Multilingual (MLC)	6 (38)	20 (31)	16 (20)	1 (6)	26%
Total	16	65	80	17	

### Responses by AN regarding their employment and education (questions 1–5, 7)

Regarding their current employer (question 1) AN answered as follows: four (25%) were employed by a university, five (31%) by a forensic institute, four (25%) by an archaeological company, and three (19%) were self-employed.

The frequency of involvement in forensic casework was queried in question 2. Here, one participant reported no interest in being involved in forensic work, four each (25% each or 75% in total) responded that they have so far not been involved in any forensic casework, they have rarely (<once a year) worked on forensic cases or they have worked on forensic cases occasionally (more than once a year), while three (19%) reported that they were involved regularly in forensic casework, which represents their main work.

Responses to the educational background (question 3) showed that the highest degree of 9 (56%) responders was PhD or equivalent, with the remaining 6 (44%) having a master’s degree or equivalent. One-quarter of the 16 AN responders had a forensic degree (question 4), while none had acquired their forensic degree in Switzerland (question 5). When asked if they would welcome the possibility of studying FA as a major degree in Switzerland (question 7), 14 (88%) responded positively.

### Responses by PO, PR, and FP regarding their knowledge of the role of FA and level of cooperation with FAs (questions 1–5, 8–9/8–10)

When PO, PR, and FP were asked if they are familiar with the role of FAs (question 1), 14% of PO, 25% of PR, and 12% of FP responded negatively, while 32% of PO, 56% of PR and 6% of FP responded they are not sure. Question 2 asked whether PO, PR and FP are aware of FAs working in Switzerland, with 95% of PO, 66% of PR and 94% of FP responding ‘yes’, while question 3 queried whether they know how to contact a FAs, with, 62% of PO, 45% of PR and 76% of FP responding positively. When FP were asked if they employed AN in their institution or whether they had any cooperation with AN (question 4, specific to FP), 35% responded that they have AN employed in their institution, 30% that they cooperate with AN, while 35% replied that they do not cooperate with AN (this response was only given by FP in DEC).

Table 2 shows the distribution of responses to questions 4 (PO, PR) and 5 (FP), which asked how often PO, PR and FP work with FAs. The most common responses were ‘never’ or ‘rarely (<1 case annually), accounting for 81% of responses from PO, 89% from PR and 70% from FP. While two-thirds of FP in FRC reported working with FA occasionally or regularly, this was the case for 10% of DEC FP.

**Table 2:** Responses of police officers, prosecutors, and forensic pathologists about how frequently they work with a FAs

*How often do you work with forensic anthropologists?*				
*Profession*	*Never*	*Rarely (<1/yr)*	*Occasionally*	*Regularly (several times/yr)*
	*n (%)*	*n (%)*	*n (%)*	*n (%)*
*Police officers*	15 (23)	38 (58)	9 (14)	3 (5)
*Prosecutors*	38 (48)	33 (41)	6 (8)	3 (4)
*Forensic pathologists*	6 (35)	6 (35)	2 (12)	3 (18)

In question 5, PO and PR were asked if they usually contact FAs directly, or if they had a different contact person in cases of skeletal remains. Of the 80 PR, 28% replied they would contact FAs directly, 20% had a different contact person and 53% had as of the time of enquiry no interaction with such cases. Among the 16 PR, who would contact someone else, 88% would contact an institute of legal medicine and 6% would contact a forensic institute. Among PO, 17% would contact FAs directly, 52% would contact someone else, and 31% had so far, no interaction with skeletal cases. Among the 34 PO, who would turn to a different contact person, 79% would contact an institute of legal medicine and 9% would contact a forensic institute. (The responses mentioning a forensic institute were all from DEC and pertained to the Zurich Forensic Science Institute.)

Question 8, specific to PO, asked what they usually do if they encounter a case concerning skeletal remains. Among the 65 responders, 12% said they do not work such cases, 2% would contact a FAs, 29% would collect the remains and bring them to FP, and 57% responded that their actions would depend on the specific case.

Questions 8–9 (PR, FP) and 9–10 (PO) queried whether PO, PR and FP have ever attended a presentation on the role of FA, and whether they would be interested in such a presentation, respectively. Overall, 25% of PO, 18% of PR, and 76% of FP responded positively (Table 3). In comparison, all FP in FRC and MLC attended a presentation on the role of FA versus 60% in DEC. In total, 88% of PO, 96% of PR and 95% of FP would be interested in a presentation on the role of FA (Table 3).

**Table 3:** Responses of police officers, prosecutors and forensic pathologists about whether they have attended, or they would be interested to attend a presentation on the role of FA

*Have you ever attended a presentation on the role of FA?*		*Would you be interested in a presentation on the role of FA?*	
*Profession*	*Yes*	*Yes*	*Yes, but only if it would take less than 2 hours*
	*n (%)*	*n (%)*	*n (%)*
*Police officers*	16 (25)	44 (68)	13 (20)
*Prosecutors*	14 (18)	50 (63)	26 (33)
*Forensic pathologists*	13 (76)	12 (71)	4 (24)

### Responses by PO, PR, FP, and AN regarding the main tasks of FAs (question 6)

**Fig. 2:**
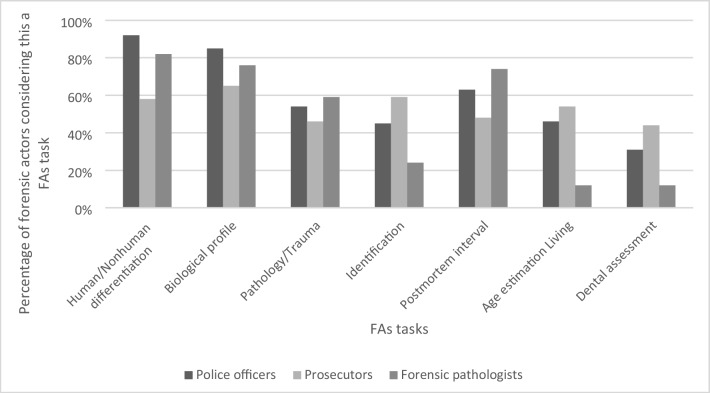
Responses of police officers, prosecutors and forensic pathologists considering certain tasks typical for forensic anthropologists (FAs)

In question 6 the AN were asked what they consider as their main tasks, while question 6 posed to PO, PR and FP queried their perception of the main tasks of FAs. The AN answered positively concerning the following main tasks: 94% establishment of biological profile, 88% assessment of skeletal trauma and pathology, 56% assistance with identification of human remains, 38% estimation of age in the living, 38% assistance with humanitarian action, and 69% research. The differentiation between human and nonhuman remains was considered a main FA task by 92% of PO, 58% of PR and 82% of FP, the establishment of biological profile by 85% of PO, 65% of PR, and 76% of FP, the assessment of skeletal trauma and pathology by 54% of PO, 46% of PR, and 59% of FP, the assistance with identification by 45% of PO, 59% of PR, and 24% of FP, the assessment of the post-mortem interval by 63% of PO, 48% of PR, and 71% of FP, the age estimation in the living by 46% of PO, 54% of PR, and 12% of FP, and dental assessment by 31% of PO, 44% of PR, and 12% of FP (Fig. 2).

### Responses by PO, PR, and FP regarding their knowledge of different methodologies associated with FA (question 7)

**Fig. 3:**
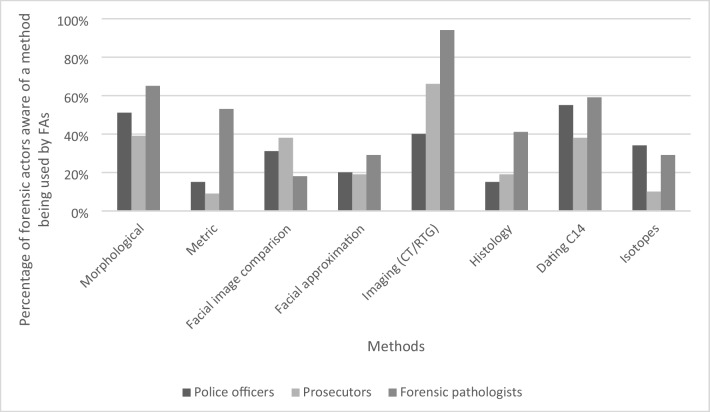
Responses of police officers, prosecutors, and forensic pathologists considering awareness about certain methods being applied by forensic anthropologists (FAs); CT: computed tomography, RTG: radiography

The responses for question 7 were grouped into four main categories: general (morphological, metric), specialized (FIC, facial approximation), technical (imaging, histology), and analytical (dating, isotopes). In this question, PO, PR, and FP were asked whether they are aware of certain methodological approaches being applied by FAs. Figure 3 shows responses by PO, PR and FP concerning the individual methods. Overall, FP were more likely to be aware of FAs using general (morphological 65% and metric assessment 53%), and technical methods (imaging 94% and histology 41%) compared with PO (51%, 15%, 40%, and 15%, respectively) and PR (39%, 9%, 66%, and 19%, respectively). In contrast, PO and PR were more aware of FIC being performed by FAs (31% and 38%, respectively) versus 18% of FP.

**Fig. 4:**
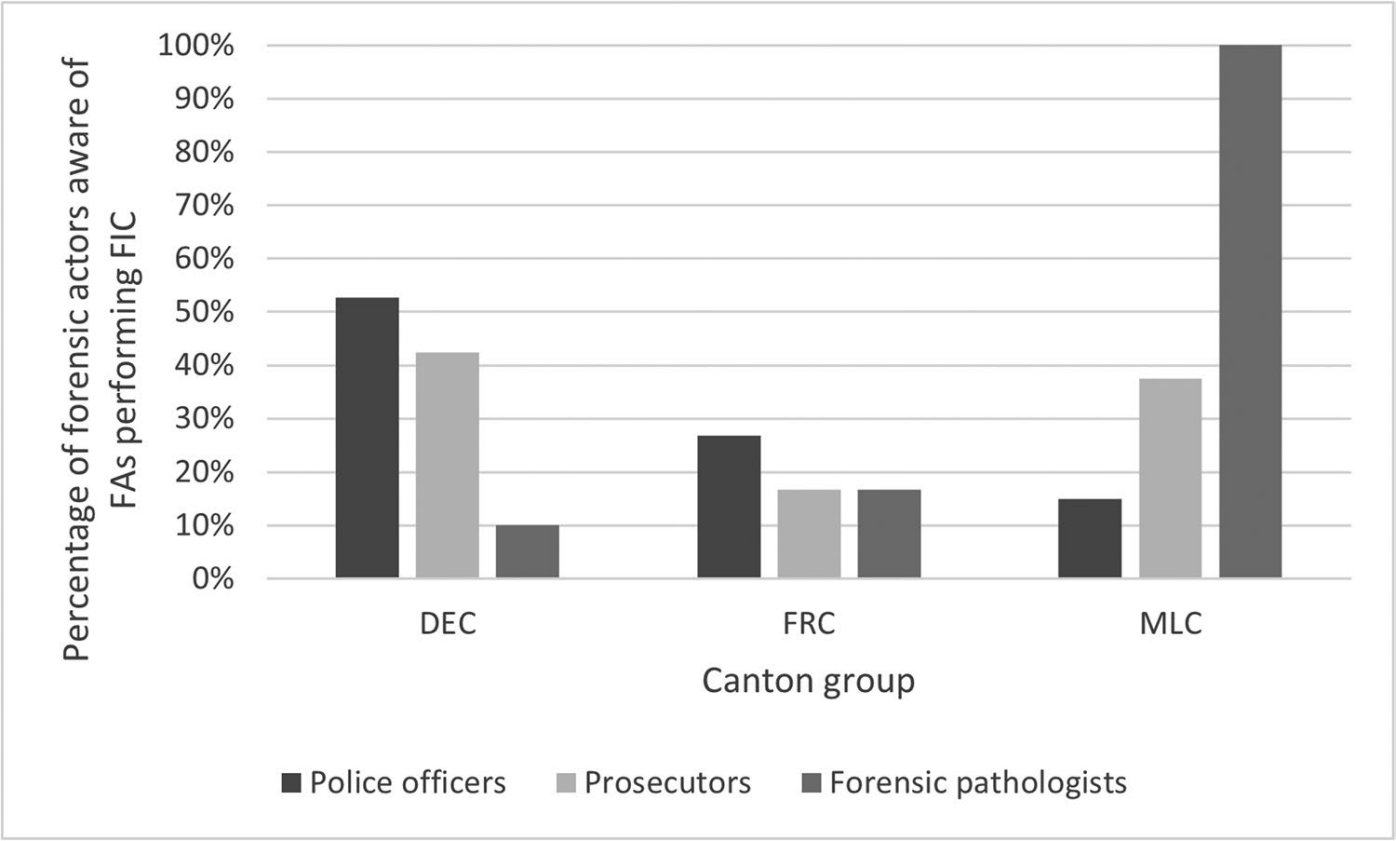
Responses of police officers, prosecutors and forensic pathologists considering awareness of facial image comparison (FIC) being performed by forensic anthropologists (FAs) by canton group

Figure 4 shows the variation of responses by canton group and profession regarding the awareness of FIC being a method used by FAs. This method was selected because it is performed by forensic anthropologists solely at the Zurich Forensic Science Institute located in DEC. Both PO and PR in DEC were more frequently (53% and 42%, respectively) aware of facial image comparison being performed by FAs compared with PO and PR in FRC (27% and 17%, respectively) and MLC (15% and 38%, respectively).

## Discussion

Forensic anthropologists have had positions at Swiss universities and governmental institutions for more than a decade, but little is known about how the role of FA is understood by different actors (police officers, prosecutors, and forensic pathologists) involved in legal proceedings regarding human identification and other aspects of forensic anthropological expertise. To address this question, a survey was designed and distributed among anthropologists and the above-mentioned forensic professionals in Switzerland. The results of this survey help identify potential knowledge gaps, which can in turn be translated into evidence-based education pathways. In addition, information about different employment and cooperation structures involving FA and the other forensic actors can guide future working environments and collaboration strategies in cases of skeletal remains and human identification not just in Switzerland but worldwide.

### Education of Swiss (forensic) anthropologists

Responses to questions about the educational and professional background of AN revealed that more than half held a PhD or equivalent, but only a quarter of the responders had forensic-specific degrees. Those degrees were acquired at foreign universities as there is currently no possibility of gaining a FA degree in Switzerland, although almost 90% of the AN would welcome the possibility of studying a major degree in the discipline in Switzerland.

The current employment status of responding AN was almost equally split among universities, forensic institutes, archaeological excavation companies and freelancing. This finding is in accordance with previous surveys, which also reported a range of different employment opportunities for FAs [1, 5, 16].

### Involvement of AN/FAs in forensic casework and collaboration among forensic actors

Only three (19%) forensic anthropologists reported working on forensic cases regularly. These forensic anthropologists worked at a forensic institute (meaning an institute of forensic sciences or institute of legal medicine) and four more AN worked on forensic cases occasionally (>1 case/annually); two reported working for a forensic institute and two for a university. The case numbers can vary greatly per year but overall, an increase in FAs’ involvement in skeletal investigations is recorded in Switzerland according to the authors and Indra and Lösch [7]. Considering this, the reported collaboration of other forensic stakeholders with FAs occasionally or regularly (19% of PO, 11% of PR and 30% of FP) is not surprising.

The cooperation patterns varied by canton group, where 67% of FP in FRC reported working with FAs occasionally or regularly, compared with 10% of FP in DEC. This may be explained by the employment strategies for FAs differing between these canton groups; with all FP in FRC responding that they employ FAs in their institution and some FP in DEC replying that they do not cooperate with FAs at all. Due to a lack of responses from FP in MLC, it was not possible to make a valid conclusion. This difference may also partly reflect the duration of established FA positions in the various regions. While FA positions in FRC have been established for more than ten years, in DEC the FAs at the FOR have taken over the responsibility for the analysis of skeletal remains from FP in 2020.

Overall, 62% of PO, 45% of PR, and 76% of FP reported that they know how to contact FAs. However, only a fraction of police officers (17%) and prosecutors (28%) reported that they would contact FAs directly should the need arise. More than half of PO and one in five PR (with more than 50% having not worked on a case, which required an interaction with FAs) would prefer contacting someone else, mostly an institute of legal medicine. When PO were asked about how they usually handle a case concerning skeletal remains, only 2% would contact FAs, more than half would tailor their action to the specific case and 29% would collect the remains and bring them to FP.

**Fig. 5:**
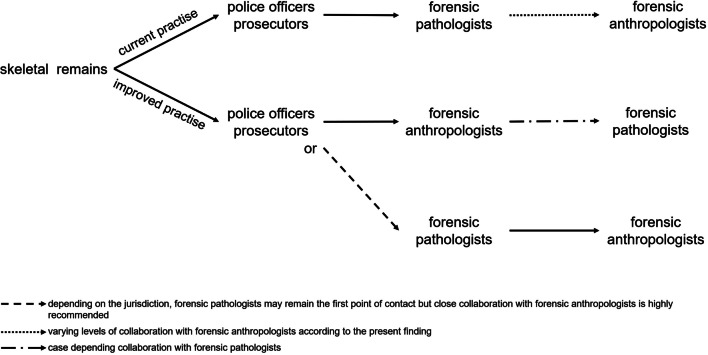
Depicting the current pathway of handling skeletal remains according to the present finding for Switzerland and an advised improved pathway optimizing the inclusion of FA knowledge in relevant casework

Ideally, the police would directly contact FAs when skeletal remains are found or FP would forward the request to FAs, seeing that they seem to be the primary contact so far (Fig. 5). However, as the results of this survey indicate, there is an overall knowledge gap about FAs working in Switzerland, how to contact them, and what their competencies are across the different forensic actors. Moreover, there are currently no standardised national guidelines on how to deal with skeletal human remains, which results in PO, who are usually first responders in such cases, acting on a case-by-case basis rather than having a standardised procedure when skeletal remains are concerned. The solution to strengthen collaborations, streamline workflows and provide the most appropriate expertise in cases of skeletal remains would be to first establish national guidelines on how to deal with skeletal human remains, and second, a structured dissemination of information about FA competencies to other forensic stakeholders. Similar recommendations were previously emphasized in international studies on FAs involvement in casework [1, 5, 8, 13, 16].

### Familiarity with the role of FAs, their competencies and methods among police officers, prosecutors and forensic pathologists

This survey found that more than 80% of Swiss FP reported being familiar with the role of FA, compared with only 54% of PO and 19% of PR. While the majority of PO and FP were aware of FAs working in Switzerland, only two-thirds of PR reported such knowledge. The different responses about the familiarity with the role of FA closely reflected the variation in positive replies regarding the attendance of a presentation on FA with 76% FP but only 18% of PR reporting ever attending a presentation on FA. Similarly, the difference in the cooperation patterns of FP and FAs found between DEC and FRC are mirrored in the differences regarding previous attendance of a presentation on FA, with all FP in FRC but only 60% of FP in DEC reporting presentation attendance.

Most respondents (88% of PO, 96% of PR and 95% of FP) would welcome a presentation on the role of FA in the future. These findings highlight that although the knowledge about FAs competencies is limited, the willingness to learn more about the discipline and how it can contribute to forensic investigations is present and should be built upon by FAs providing lectures, seminars and other educational opportunities about their field of work. Moreover, close cooperation (especially between FAs and FP) clearly fosters an understanding of the respective roles.

The responses regarding the main tasks of FA can be interpreted in light of FA-specific literature, assigning an expected value of 100% to tasks, which are typically addressed in FA textbooks (e.g. [17–20]) or have been identified as main tasks in other FA surveys/studies [4, 5, 9, 12, 14, 21, 22]. These tasks include the evaluation of human vs. non-human bones, the assessment of skeletal features leading to biological profile, the descriptive evaluation of skeletal trauma and pathology, and the assistance in the identification process of human remains. Other tasks that have been associated with FA but are either country-/laboratory-specific or are considered emerging areas in FA were assigned partially arbitrary expected percentages following the results of recent surveys [1, 5]. These tasks, with expected percentages noted in brackets, were the evaluation of the post-mortem interval (75%), age estimation of the living (50%), and dental assessment (20%). It should be noted that these percentages represent a reflection of mixed, different wholes, such as how often they are performed in different countries by FAs (in some this may be 100%, while in others 0%), how often they are performed by forensic anthropologists (some may perform these tasks on a daily basis, while others rarely or never), or whether these are perceived as FA tasks (some may be perceived as such but may never be performed by FAs).

Table 4 shows the comparison between the expected and the survey responses regarding FA competencies. The responses of AN reflected well the expected values regarding the establishment of biological profile, and the assessment of skeletal trauma and pathology (note: they were not asked about human/nonhuman differentiation, post-mortem interval and dental assessment). However, only 56% of AN were considered assistance with the identification of human remains as their task. This surprising result may be explained by the fact that only a quarter of the responding AN had a forensic-specific degree and a quarter have not been involved in forensic casework at all. Although similar methods are used in biological and forensic anthropology, the objectives of the subdisciplines differ as Passalacqua et al. [10] recently argued that forensic anthropology should no longer be considered a sub-discipline of bioarchaeology (biological anthropology) as the responsibilities and tasks of FA diverted into specialized and distinct requests closely related to forensic sciences.

**Table 4:**
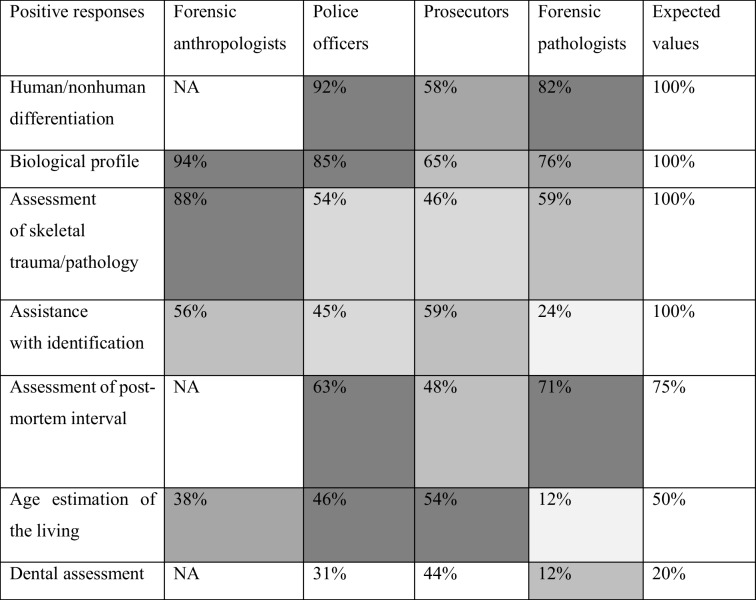
The comparison of the percentage of positive responses and expected values concerning the perception of main FA tasks among the various professions. The darker the fill, the closer the response to the expected value. The increment of greyscale difference represents -15% from the expected value, e.g., the initial value of 50% has the following increments: 42.5%, 35%, 27.5%, 20%, 12.5% and 5%. No fill is assigned to values that exceed the expected value by more than 5%

The response of PO aligned well with the expected values for human/nonhuman differentiation, biological profile, estimation of post-mortem interval and age estimation of the living, while they exceeded the expected value in dental assessment and only half of the PO viewed skeletal trauma/pathology assessment and identification as FA tasks. Prosecutors have only considered age estimation of the living in agreement with the expected value, while their response exceeded the expected value for dental assessment and was lower than 30% of the initial value for human/nonhuman differentiation, biological profile, assessment of skeletal trauma/pathology, post-mortem interval, and assistance with identification. These values seem to reflect the lack of awareness of PR about the role of FA as shown in their reported responses throughout this survey. Further education for PR, which they reported to be welcome, should therefore be a priority, considering that they commission forensic experts.

The anthropologists were additionally asked if they considered assistance with humanitarian action and research as one of the main tasks, with positive responses of about one-third and two-thirds, respectively. Since FA in humanitarian actions and disaster victim identification is an emerging field [23–25] it is not surprising that a relatively low number accounted for this as a main task of FAs (38%). Even though conducting research is not requested at every FA employment, research is significant for the discipline and positive responses would have been expected to be higher (69%). This discrepancy may be explained by the varied employment situations of the responding AN (e.g., a quarter were freelancers) and possibly the fact that research might not be part of employment contracts, as well as limited time, money and/or access to specimens, which might hinder research in the field to be considered.

The survey concluded by asking about the awareness of PO, PR and FP regarding methodological approaches used by FAs. The knowledge of general methods (morphological and metric), which are routinely used by FAs for the assessment of human/nonhuman origin, biological profile, and identification, was surprisingly limited, ranging between 9% of PO for metrics and 65% in FP for morphology. Overall, FP were more likely to be familiar with the methodological approaches of FA than PO and PR, except for FIC. However, FIC was registered by about one-third of PO and PR, although this increased to almost 50% in DEC, where this specialized methodology is applied routinely by FAs and trained experts employed at the Zurich Forensic Science Institute. It is not surprising that FP are less aware of FIC than PO and PR as the latter two professionals are mainly give mandates in such cases.

A limitation of this study is the underrepresentation of responses from DEC AN and PO, as well as MLC FP. The reasons for the observed response rate are difficult to evaluate as the survey was distributed via e-mail to the different working groups with the request to pass the link to the relevant personnel. This, however, relies on the goodwill of the first in line to pass the survey on as well as on the participants to spare time to respond to a survey that relates to the highly specialized discipline of forensic anthropology. Nevertheless, the high participation rate of PR of the various canton shows the interest of the important representatives of the cantonal government in criminal cases. However, the results of this questionnaire showed that the competencies and skills of FAs are often not well understood among forensic stakeholders. This survey indicates that in the future, FAs in Switzerland should establish structured and close collaboration with PO, PR and FP, provide more insights into FA to the various professions, and finally continue their own professional training.

## Conclusion

To our knowledge, this is the first study addressing the perception of forensic anthropology among related professions, including police officers and prosecutors. This survey showed inherent gaps in the knowledge of the role and competencies of FAs among police, prosecutors, and forensic pathologists in Switzerland. However, an overwhelming majority of the participants indicated willingness to learn more about the discipline of FA and therefore it is recommended that forensic anthropologists promote their field of expertise by disseminating information about FA tasks and competencies in lectures or seminars for other forensic actors. While FAs have been working in Switzerland for over a decade, it seems that establishing such a specialized discipline in a country with multiple official languages and a variety of local laws and regulations may be a slow process, where close collaboration networks (e.g., forensic anthropologists being employed at institutions with forensic pathologists or the police) lead to improved understanding of the benefits of FA in forensic investigations.

By exploring the perception of the role of FA among other forensic actors in Switzerland, a country with strong cantonal division in laws and language, the findings of this survey may be extrapolated to European or even global level in relation to the optimization of employment structures and educational pathways in forensic disciplines.

### Supplementary information

ESM 1 (DOCX 24 kb)

## Data Availability

The data presented in this study are available on request from the corresponding author. The data are not publicly available due to ethical restrictions.
