# Supplementary material for: Awareness of forensic anthropology in Switzerland: a survey among forensic practitioners, police, and prosecutors
Source: Int J Legal Med. 2023 Nov 15;138(3):1067–77. doi: 10.1007/s00414-023-03116-9 (PMC11003926; doi:10.1007/s00414-023-03116-9)
Supplement: Supplementary file 1 — (DOCX 24 kb) [file 414_2023_3116_MOESM1_ESM.docx]

# Supplementary material 1 – Questionnaires

The original versions of the questionnaires were written/distributed in three languages (French, German, Italian). The questionnaire was translated to English for the sake of this publication.

## General

All participants were asked:

1. to select their preferred language (German, French, Italian)
2. indicate their profession (this directed them to the main survey)
3. to select the Canton of their main employment

## Anthropologists (AN)

1. *As an anthropologist in Switzerland, indicate your main employer*:

a. a university

b. a forensic centre (which may or may not be affiliated to a university)

c. commercial archaeology

d. cantonal services

e. independent anthropologist

2. *How often are you asked to participate in forensic cases, including initial assessment of human vs non-human bones*?

a. On a regular basis, it’s my main work priority

b. From time to time, but definitively more than once a year

c. Rarely, maybe once every few years

d. I have never been asked to participate in a forensic case

e. I do not want to work in forensic cases

3. *What is your highest degree*?

a. Bachelor’s degree

b. Master’s degree

c. PhD degree

d. none of the above, please specify:

4. *Was one or any of your degrees specifically forensic (e.g. Master in forensic archaeology and/or anthropology)?*

a. No

b. Yes

5. *If you have a specific forensic degree, was it acquired in Switzerland?*

a. Yes

b. No. Please, specify country issuing the degree:

c. I don’t have a specific forensic degree

6. *What do you think of as the main task(s) of forensic anthropologists (more than one answer is possible)*?

a. Establishing biological profile of skeletal remains

b. Evaluating bone trauma in forensic cases

c. Visual identification of persons and other identifications

d. Age estimation in living individuals

e. Forensic humanitarian action

f. Research

g. Others, specify:

7. *Do you think Switzerland needs its own forensic anthropology degree course (Bachelor, Master, Doctorate programs)*?

a. Yes. Please, explain your answer:

b. No. Please, explain your answer:

8. *Would you like to leave us a comment about forensic anthropology in Switzerland, an suggestion for the future, or a comment about this study*?

## Forensic Pathologists (FP)

1. *Are you familiar with the capabilities of forensic anthropologists*?

a. Yes

b. No

c. I am not sure

2. *Do you know if there are any forensic anthropologists working in Switzerland*?

a. Yes

b. No

*3. Do you know how to contact a Swiss forensic anthropologist*?

a. Yes

b. No, and I do not need to know

c. No, but I would like to have that information

4. *Does your institute employ forensic anthropologists*?

a. Yes, fulltime

b. Yes, but only on casual basis/case-specific

c. Not employed, but we collaborate with forensic anthropologists

d. Not that I know of

5. *As a forensic pathologist, how often do you have collaborative cases with forensic anthropologists*?

a. Several times per year

b. Once or twice a year

c. Rarely. Once every few years

d. Never

6. *What are your expectations of forensic anthropologists if you would request them (more than one response is possible)?*

a. To identify a person (whether dead or alive)

b. To provide information on trauma and other pathologies and their timing

c. To estimate the age of living individuals

d. To estimate how long it has been since an individual died

e. To distinguish whether skeletal remains are human or nonhuman

f. To estimate the age, sex, and stature of skeletal remains

g. To assess dental profiles for identification

h. Other, specify:

7. *Among the techniques that can be used by a forensic anthropologist, indicate the one(s) you are familiar with (more than one response is possible)*:

a. Morphological analysis

b. Metric analysis

c. Radiocarbon dating

d. Isotope analysis

e. Facial reconstruction

f. CT scanning

g. Visual identification of persons (image-to-image comparison, facial image comparison)

h. Histological analysis

8. *During your career, have you ever attended a presentation/seminar about the role and possibilities of forensic anthropology*?

a. Yes

b. No

9. *If such a presentation would be proposed to your office, would you be willing to attend*?

a. Yes, whatever the duration will be

b. Yes, but only if it will take less than 2 hours

c. No thank you

d. No, but I would like to get some contact details

10. *Would you like to leave us a comment about forensic anthropology in Switzerland, an suggestion for the future, or a comment about this study*?

## Prosecutors (PR)

1. *Are you familiar with the capabilities of forensic anthropologist*?

a. Yes

b. No

c. I am not sure

2. *Do you know if there are any forensic anthropologists working in Switzerland*?

a. Yes

b. No

3. *Do you know how to contact a Swiss forensic anthropologist*?

a. Yes

b. No, and I do not need to know

c. No, but I would like to have that information

4. *As a prosecutor in Switzerland, how often do you have collaborative cases with forensic anthropologists*?

a. Several times per year

b. Once or twice a year

c. Rarely. Once every few years

d. Never

5. *Do you usually deal with forensic anthropologists directly?*

a. I deal directly with the anthropologist

b. I am usually in touch with someone else; specify:

c. None of the above, I don’t work with forensic anthropologists

6. *What are your expectations of forensic anthropologists if you would request them (more than one response is possible?*

a. To identify a person (whether dead or alive)

b. To provide information on trauma and other pathologies and their timing

c. To estimate the age of living individuals

d. To estimate how long it has been since an individual died

e. To distinguish whether skeletal remains are human or nonhuman

f. To estimate the age, sex, and stature of skeletal remains

g. To assess dental profiles for identification

h. Other, specify:

7. *Among the techniques that can be used by a forensic anthropologist, indicate the one(s) you are familiar with (more than one response is possible)*:

a. Morphological analysis

b. Metric analysis

c. Radiocarbon dating

d. Isotope analysis

e. Facial reconstruction

f. CT scanning

g. Visual identification of persons (image-to-image comparison, facial image comparison)

h. Histological analysis

8. *During your career, have you ever attended a presentation/seminar about the role and possibilities of forensic anthropology*?

a. Yes

b. No

9. *If such a talk would be proposed to your office, would you like to attend*?

a. Yes, whatever the duration will be

b. Yes, but only if it took less than 2 hours

c. No thank you

d. No, but I would like to get some contact details

10. *Would you like to leave us a comment about forensic anthropology in Switzerland, an suggestion for the future, or a comment about this study*?

## Police Officers (PO)

1. *Are you familiar with the capabilities of forensic anthropologist*?

a. Yes

b. No

c. I am not sure

2. *Do you know if there are any forensic anthropologists in Switzerland*?

a. Yes

b. No

3. *Do you know how to contact a Swiss forensic anthropologist*?

a. Yes

b. No, and I do not need to know

c. No, but I would like to have that information

4. *As a member of the police in Switzerland, how often do you interact with forensic anthropologists on your cases*?

a. Several times per year

b. Once or twice a year

c. Rarely. Once every few years

d. Never

5. *Do you usually deal with forensic anthropologists directly?*

a. I deal directly with the anthropologist

b. I am usually in touch with someone else; specify:

c. None of the above, I don’t work with forensic anthropologists

6. *What are your expectations of forensic anthropologists if you would request them (more than one response is possible)?*

a. To identify a person (whether dead or alive)

b. To provide information on trauma and other pathologies and their timing

c. To estimate the age of living individuals

d. To estimate how long it has been since an individual died

e. To distinguish whether skeletal remains are human or nonhuman

f. To estimate the age, sex, and stature of skeletal remains

g. To assess dental profiles for identification

h. Other, specify:

7. *Among the techniques that can be used by a forensic anthropologist, indicate the one(s) you are familiar with (more than one response is possible)*:

a. Morphological analysis

b. Metric analysis

c. Radiocarbon dating

d. Isotope analysis

e. Facial reconstruction

f. CT scanning

g. Visual identification of persons (image-to-image comparison, facial image comparison)

h. Histological analysis

8. *When you are facing the findings of bones, do you*:

a. Call the anthropologists to come take a look

b. Just bring the bones to the legal medicine centre

c. Depends on the situation

d. I don’t deal with bones

9. *During your career, have you ever attended a presentation/seminar about the role and possibilities of forensic anthropology*?

a. Yes

b. No

10. *If such a talk would be proposed to your office, would you like to attend*?

a. Yes, whatever the duration will be

b. Yes, but only if it took less than 2 hours

c. No thank you

d. No, but I would like to get some contact details

11. *Would you like to leave us a comment about forensic anthropology in Switzerland, an suggestion for the future, or a comment about this study*?
